# PDGF Upregulates Mcl-1 Through Activation of β-Catenin and HIF-1α-Dependent Signaling in Human Prostate Cancer Cells

**DOI:** 10.1371/journal.pone.0030764

**Published:** 2012-01-20

**Authors:** Shareen Iqbal, Shumin Zhang, Adel Driss, Zhi-Ren Liu, Hyeong-Reh Choi Kim, Yanru Wang, Chad Ritenour, Haiyen E. Zhau, Omer Kucuk, Leland W. K. Chung, Daqing Wu

**Affiliations:** 1 Department of Urology and Winship Cancer Institute, Emory University School of Medicine, Atlanta, Georgia, United States of America; 2 Department of Microbiology, Biochemistry and Immunology, Morehouse School of Medicine, Atlanta, Georgia, United States of America; 3 Department of Biology, Georgia State University, Atlanta, Georgia, United States of America; 4 Department of Pathology, Barbara Ann Karmanos Cancer Institute, Wayne State University, School of Medicine, Detroit, Michigan, United States of America; 5 Uro-Oncology Research Program, Department of Medicine, Cedars-Sinai Medical Center, Los Angeles, California, United States of America; 6 Department of Hematology and Medical Oncology and Winship Cancer Institute, Emory University School of Medicine, Atlanta, Georgia, United States of America; Roswell Park Cancer Institute, United States of America

## Abstract

**Background:**

Aberrant platelet derived growth factor (PDGF) signaling has been associated with prostate cancer (PCa) progression. However, its role in the regulation of PCa cell growth and survival has not been well characterized.

**Methodology/Principal Findings:**

Using experimental models that closely mimic clinical pathophysiology of PCa progression, we demonstrated that PDGF is a survival factor in PCa cells through upregulation of myeloid cell leukemia-1 (Mcl-1). PDGF treatment induced rapid nuclear translocation of β-catenin, presumably mediated by c-Abl and p68 signaling. Intriguingly, PDGF promoted formation of a nuclear transcriptional complex consisting of β-catenin and hypoxia-inducible factor (HIF)-1α, and its binding to Mcl-1 promoter. Deletion of a putative hypoxia response element (HRE) within the Mcl-1 promoter attenuated PDGF effects on Mcl-1 expression. Blockade of PDGF receptor (PDGFR) signaling with a pharmacological inhibitor AG-17 abrogated PDGF induction of Mcl-1, and induced apoptosis in metastatic PCa cells.

**Conclusions/Significance:**

Our study elucidated a crucial survival mechanism in PCa cells, indicating that interruption of the PDGF-Mcl-1 survival signal may provide a novel strategy for treating PCa metastasis.

## Introduction

The platelet-derived growth factors (PDGF) family consists of five dimeric isoforms: PDGF-AA, -AB, -BB, -CC and -DD [1], which exert their cellular effects through two structurally similar tyrosine kinase receptors (PDGFR-α and -β) expressed by many different cell types [2]. Ligand binding to PDGFRs results in the dimerization and autophosphorylation of the receptor kinases, subsequently recruiting certain Src homology 2 (SH2) domain-containing adaptor proteins (e.g., Src, Grb2 and Shc) to specific phosphorylated tyrosine residues. Several signaling cascades, including Ras-mitogen-activated protein kinase (MAPK), phospholipase-γ and phos-phatidyl-inositol-3′-kinase (PI3K)/Akt, have been characterized as the major downstream pathways mediating PDGF functions [3]. Other adaptor molecules (e.g., the Fer and Fes tyrosine kinase family) and transcriptional factors (e.g., β-catenin) are also involved in PDGF signaling in certain cell types [4], [5], [6], [7], [8].

Aberrant PDGF expression has been frequently associated with the neoplastic component of human tumors, whereas PDGFRs are mainly found in the fibroblastic and vascular tumor stroma [9], [10], [11], [12], [13]. These observations suggested that tumor-derived PDGF may primarily act as a paracrine signaling molecule in solid tumors. Supporting this concept, recent studies have demonstrated that PDGF is a potent pro-angiogenic factor by promoting the recruitment and growth of stromal fibroblasts, perivascular cells and endothelial cells, thereby indirectly affecting tumor growth, metastatic dissemination and drug resistance [2], [3], [14], [15], [16]. Interestingly, emerging evidence indicated that PDGF autocrine signaling may also play an important role during tumor progression. Mutational activation or co-expression of PDGF ligands and receptors are capable of stimulating tumor cell growth and proliferation in several nonepithelial malignancies, including glioblastomas and osteosarcoma [17]. More recently, autocrine PDGF signaling has been associated with epithelial-to-mesenchymal transition (EMT) in carcinoma cells from the breast, colon, prostate and liver, suggesting a causative role of autocrine PDGF signaling in metastasis [7], [8], [18], [19]. Nonetheless, despite the well-established correlation between deregulated paracrine PDGF signaling and tumor progression, the functions and mechanisms of autocrine PDGF signaling in epithelial cancer cells remain elusive [3], [17].

Acquisition of apoptosis resistance is characteristic of metastatic tumor cells, which may confer survival advantages during invasion, metastasis and colonization [20]. We recently correlated overexpression of myeloid cell leukemia-1 (Mcl-1), a member of the Bcl-2 family, with the progression of prostate cancer (PCa) towards bone metastasis [21]. In this study, we provide evidence that PDGF-BB is a survival factor in metastatic PCa cells by upregulating Mcl-1 expression through a signaling mechanism mediated by the transcriptional factors β-catenin and hypoxia-inducible factor (HIF)-1α.

## Materials and Methods

### Cell Culture

Human PCa cell lines ARCaP_E_, ARCaP_M_ [22], LNCaP (American Type Culture Collection, ATCC, Manassas, VA), C4-2 [23] and PC3 (ATCC) were routinely maintained in T-medium (Invitrogen, Carlsbad, CA) with 5% fetal bovine serum (FBS). For the treatments with PDGF isoforms, PCa cells seeded in 96-well plates (3,000 cell/well) were serum-starved overnight, replaced with fresh serum-free T-medium, and incubated in the presence of varying concentrations of recombinant human PDGF-AA, -AB, -BB (R&D Systems, Minneapolis, MN), or phosphate-buffered saline (PBS) for indicated times. Recombinant human interleukin-6 (IL-6) was purchased from R&D Systems. For chemotherapy drug treatment, docetaxel (Sanofi Aventis, Bridgewater, NJ) or dimethyl sulfoxide (DMSO; Sigma-Aldrich, St. Louis, MO) was added to cells and incubated for 72 h. Cell proliferation was measured using the CellTiter 96 AQ proliferation assay according to the manufacturer's instructions (Promega, Madison, WI). Viable cells were counted in triplicate using a hemacytometer and trypan blue staining.

### Plasmids and small interfering RNAs (siRNAs)

The full-length human Mcl-1 promoter region cloned into a firefly luciferase reporter vector pGL3-Basic (Promega, Madison, WI) was kindly provided by Dr. Steven W. Edwards (University of Liverpool, Liverpool, UK) [24]. The hypoxia-responsive element (HRE) (fragment −900 to −884)-truncated construct was obtained by digestion of the full-length promoter using KpnI (from position −3914 to −855) and then ligated using T4 DNA ligase (New England Biolabs, Ipswich, MA). Both plasmid constructs were confirmed by sequence analysis. The pHIF1-luc reporter was purchased from Panomics (Fremont, CA). TOPFlash and FOPFlash T-cell factor (TCF) reporters were obtained from Upstate (Billerica, MA). pTK-RL plasmid was purchased from Promega. Human Mcl-1 expression vector (pCMV-Mcl-1) was obtained from Origene, Inc. Human β-catenin expression plasmid was provided by Dr. Zhi-Ren Liu. ON-TARGET*plus* SMARTpool siRNAs against β-catenin, p68, PDGFR-α and PDGFR-β, and control siRNA were obtained from Dharmacon, Inc (Chicago, IL). HIF-1α and control siRNA were purchased from Santa Cruz Biotechnology, Inc. (Santa Cruz, CA). Transient transfection of DNA constructs and siRNAs was performed using Lipofectamine 2000 or Oligofectamine reagents (Invitrogen), according to the manufacturer's protocols and our published procedures [21], [25].

### Western Blot Analysis

Total cell lysates were prepared using radioimmunoprecipitation (RIPA) buffer (Santa Cruz Biotechnology, Inc.). Nuclear proteins were extracted using a Novagen kit (EMD Biosciences, San Diego, CA). Immunoblotting analysis followed standard procedures [25]. ImageJ software (National Institutes of Health) was used to quantitate the relative protein expression as normalized to the loading controls. Information for the antibodies used in this study was described in Supplemental Table S1.

### Immunoprecipitation

The Immunoprecipitation Starter Pack (GE Healthcare Bio-Sciences Corp., Piscataway, NJ) was used according to the manufacturer's instructions. Total nuclear lysates (1 mg) were immunoprecipitated with 5 µg rabbit anti-HIF-1α antibody, mouse anti-β-catenin antibody, mouse anti-c-Abl and rabbit anti-p68 antibody (Supplemental Information, Table S1), or normal IgG (R&D Systems). Protein A/G Sepharose 4 Fast Flow beads were added to precipitate proteins, then washed and eluted. The samples were further processed for Western blot analysis.

### Immunofluorescence and Confocal Imaging

Immunofluorescence was performed as described previously [21] using mouse anti-β-catenin, rabbit anti-p68 and anti-HIF-1α antibodies (Supplemental Table S1). Either Alexa 488 or 555 secondary antibodies (Invitrogen) were used at a dilution of 1∶500 and were incubated for 1 h at room temperature. Nuclear staining was performed by incubating cells with 0.4 µmol/L 4′,6-diamidino-2-phenylindole (DAPI) to mounting slides. Cells were imaged on a Zeiss LSM 510 META. In all cases, either a 63x or 100x Zeiss Plan-Apo oil objective was used (numerical aperture of 1.3 and 1.4, respectively). All images had contrast expansion performed in Adobe Photoshop.

### Quantitative RT-PCR (qRT-PCR) and RT-PCR

Total RNA was prepared with Qiagen RNeasy Kit (Valencia, CA). The first-strand cDNA was synthesized using SuperScript ®III First-Strand Synthesis System (Invitrogen). Quantitative PCR was performed by the LightCycler 480 system (Roche Applied Science) using a Brilliant® SYBR® Green QPCR Master Mix (Stratagene) according to the manufacturer's instructions. For end-point RT-PCR, the SuperScript® III One-Step RT-PCR kit (Invitrogen) was used following the manufacturer's protocol. The specific primer pairs are described in Supplemental Table S2. Glyceraldehyde-3-phosphate dehydrogenase (GAPDH) mRNA was amplified with a pair of primers described previously [25] and used to normalize RNA inputs.

### Chromatin Immunoprecipitation Assay (ChIP)

The sequential ChIP (ChIP-re-ChIP) experiment was performed using the Active Motif Re-ChIP-IT® kit (Active Motif, Carlsbad, CA). Briefly, PCa cells were serum-starved overnight and replaced with fresh serum-free medium, incubated with PDGF-BB or PBS for indicated times. Cells were fixed 10 min at room temperature by 1% formaldehyde solution to cross-link DNA-protein interactions. Chromatin was sheared for 8 min using a ChIP-IT Express Enzymatic Shearing kit (Active Motif) [25]. A portion of chromatin was reversed and used as input DNA. For immunoprecipitation, 2 µg of anti-β-catenin antibody were added and incubated overnight, with normal IgG as the control (Supplemental Table S1). Eluted chromatin was desalted, and an aliquot was used as control for the first ChIP reaction. The re-ChIP reaction was then performed with 2 µg of anti-HIF-1α antibody or with IgG control. PCR primers for the HRE region in human Mcl-1 promoter were described in the Supplemental Table S2. PCR reactions were performed for 40 cycles, with primer concentration as 10 pmol/20 µl, using AmpliTaq Gold 360 Master Mix kit (Invitrogen).

### Statistical Analysis

All data represent three or more experiments. Errors are S.E. values of averaged results. For each assay Student's *t-test* was used for statistical comparison with the control groups. Values of *p*≤0.05 were taken as a significant difference between means. Statistical analysis was performed using the Sigmaplot software 11.0 (Systat Software, Inc., Chicago, IL).

## Results

### Mcl-1 is a survival factor in PCa cells

**Figure 1:**
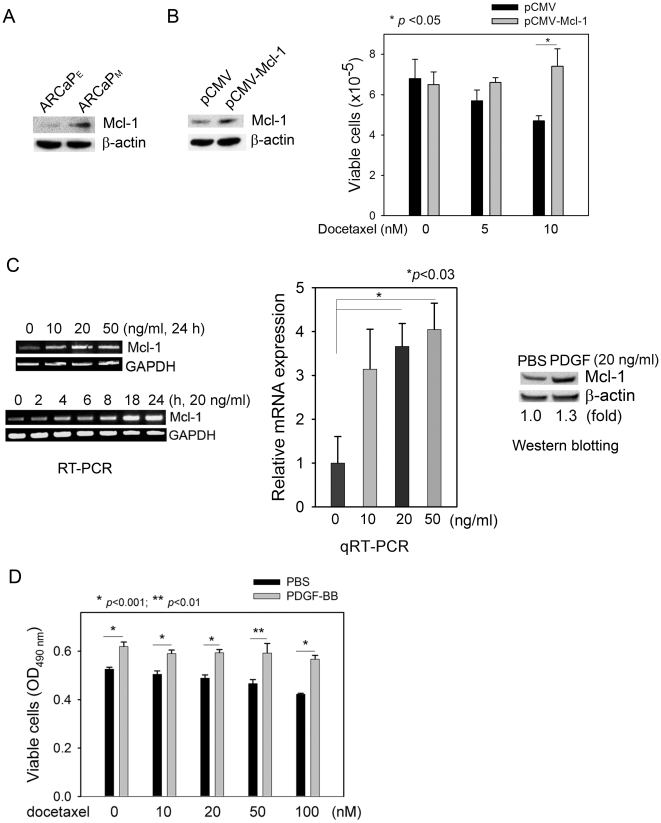
PDGF-BB upregulates Mcl-1 and protects PCa cells from apoptosis. (A) Mcl-1 protein expression in the lineage-related ARCaP_E_ and ARCaP_M_ cells. (B) Ectopic expression of Mcl-1 in ARCaP_M_ cells and the effects on docetaxel cytotoxicity in ARCaP_M_ cells. pCMV: vector control. (C) Left panel: The dose- and time-dependent effects of PDGF-BB on Mcl-1 mRNA expression in ARCaP_M_ cells; middle panel: qRT-PCR analysis of Mcl-1 mRNA expression in response to PDGF-BB treatment in ARCaP_M_ cells (24 h); right panel: The effects of PDGF-BB treatment (20ng/ml, 72 h) on Mcl-1 protein expression in ARCaP_M_ cells. ImageJ was used to quantitate the relative expression of Mcl-1 protein. (D) The effects of exogenous PDGF-BB on the cytotoxicity of docetaxel in ARCaP_M_ cells, as determined by the MTS assay.

Previously we demonstrated that Mcl-1 overexpression is associated with *in vivo* bone metastatic propensity of human PCa cells, and importantly, correlated with clinical PCa bone metastasis [21]. Consistently, using a human PCa ARCaP cellular model that could closely mimic the pathophysiology of bone metastasis in immunocompromised mice [26], we found that Mcl-1 expression was significantly increased in highly bone metastatic ARCaP_M_ cells when compared to that in the low-invasive counterpart ARCaP_E_ cells (Figure 1A). We hypothesized that upregulation of Mcl-1 may confer metastatic PCa cells survival advantages, allowing them to escape apoptotic fate during invasion and dissemination and successfully establish distant metastasis [20]. Supporting this notion, ectopic expression of Mcl-1 enhanced PCa cell resistance to docetaxel (Figure 1B), a commonly used chemotherapeutic drug in hormone-refractory and metastatic PCa [27]. These results indicated that upregulation of Mcl-1 may account for, at least in part, resistance to apoptosis in metastatic PCa cells.

### PDGF-BB induces Mcl-1 expression and antagonizes apoptosis in PCa cells

Intriguingly, PDGF-BB was found to significantly induce Mcl-1 expression in PCa cells (Figure 1C, Supplemental Figure S1). Treatment with recombinant human PDGF-BB increased Mcl-1 mRNA in a dose- and time-dependent manner, though the optimal conditions for the maximum accumulation of Mcl-1 mRNA varied in different PCa cell lines. Western blot analysis confirmed the inductive effects of PDGF-BB on Mcl-1 expression at protein level. These data identified PDGF-BB as a novel regulator of Mcl-1 expression, which could provide a survival mechanism to protect PCa cells from apoptosis. Indeed, addition of PDGF-BB in PCa cell cultures effectively antagonized the cytotoxicity of docetaxel over a wide range of doses (Figure 1D).

### Expression profile of PDGF autocrine signaling components in PCa cells

**Figure 2:**
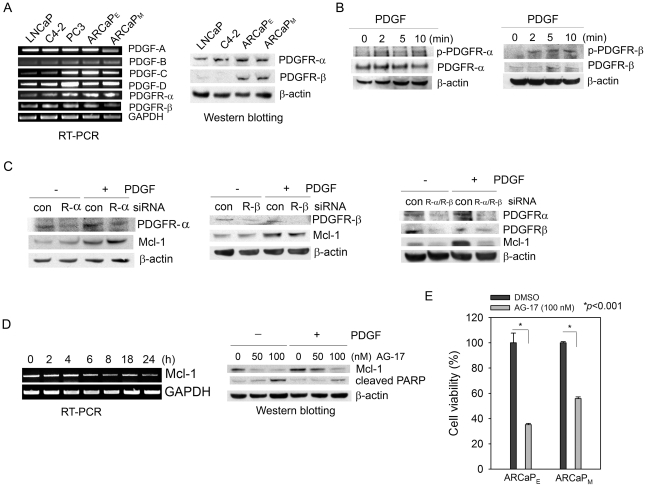
Activation of the PDGFR signaling is required for Mcl-1 expression in PCa cells. (A) Expression profile of PDGFR signaling components in PCa cells, as analyzed by RT-PCR and Western blotting. (B) The effects of PDGF-BB (20 ng/ml) on the phosphorylation of PDGFR-α and -β in ARCaP_M_ cells. (C) The effects of depleting PDGFR-α or/and -β on Mcl-1 protein expression in ARCaP_M_ cells. The cells were transfected with either isotype-specific siRNAs targeting PDGFR-α (left panel, 30 nM) or PDGFR- β (central panel, 100 nM), or a mixture of PDGFR-α and -β siRNAs (right panel) for 48 h, serum-starved overnight, and incubated in the presence or absence of PDGF-BB (20 ng/ml) for 72 h. (D) Upper panel: The time-dependent effects of AG-17 (100 nM) on Mcl-1 mRNA expression in ARCaP_M_ cells; bottom panel: The effects of AG-17 treatment on the expression of Mcl-1 and cleaved PARP in the presence (20 ng/ml) or absence of PDGF-BB (20 ng/ml) in ARCaP_M_ cells. (E) The effects of AG-17 treatment (100 nM, 72 h) on the viability of ARCaP_E_ and ARCaP_M_ cells.

We examined the expression pattern of PDGFs and their receptors in PCa cells (Figure 2A). RT-PCR analyses showed that the PDGF isoforms were differentially expressed at mRNA level, and among them, increased PDGF-B and PDGF-D were observed in C4-2 and ARCaP_M_ cells when compared to the parental LNCaP and ARCaP_E_ cells, respectively. Consistent with previous studies [19], PC3 cells were found to express high levels of PDGF-D, PDGFR-α and -β. Interestingly, PDGFR-α mRNAs appeared to be substantially expressed in PCa cells, which was confirmed at protein level by Western blot analysis. In contrary, though PDGFR-β mRNAs were detected by RT-PCR in most PCa cell lines, immunoblotting analysis could only confirm protein expression in ARCaP_E_ and ARCaP_M_ cells (Figure 2A, right panel). Taken together, these data suggested a functional PDGF autocrine signaling in certain PCa cells.

### An autocrine PDGFR signaling mediates PDGF-BB regulation of Mcl-1 in PCa cells

Both PDGFR-α and -β were highly expressed in bone metastatic ARCaP_M_ cells, and rapidly phosphorylated in a time-dependent manner in response to the stimulation of exogenous PDGF-BB (Figure 2B). Interesting, depletion of either PDGFR-α or -β by isoform-specific siRNA did not block the inductive effect of PDGF-BB on Mcl-1 expression (Figure 2C, left and central panels), suggesting that activation of either receptors may be sufficient for the upregulation of Mcl-1. Supporting this hypothesis, transient transfection with a mixture of siRNAs targeting both PDGFR-α and -β inhibited the basal expression of Mcl-1, and abrogated PDGF-BB induction of Mcl-1 ARCaP_M_ cells (Figure 2C, right panel). Alternatively, treatment with AG-17 (Tyrphostin), a selective pharmacological inhibitor of PDGFRs [28], reduced Mcl-1 expression at both mRNA and protein levels and markably increased cleavage of poly-ADP ribose polymerase (PARP), an indicator of apoptosis. These effects were attenuated by the presence of PDGF-BB in cultures (Figure 2D). Consistently, AG-17 treatment at low doses (such as 100 nM) effectively induced apoptosis in ARCaP_E_ and ARCaP_M_ cells (Figure 2E), indicating a pivotal role of PDGFR signaling in the survival of PCa cells.

### β-catenin mediates PDGF regulation of Mcl-1 expression in PCa cells

**Figure 3:**
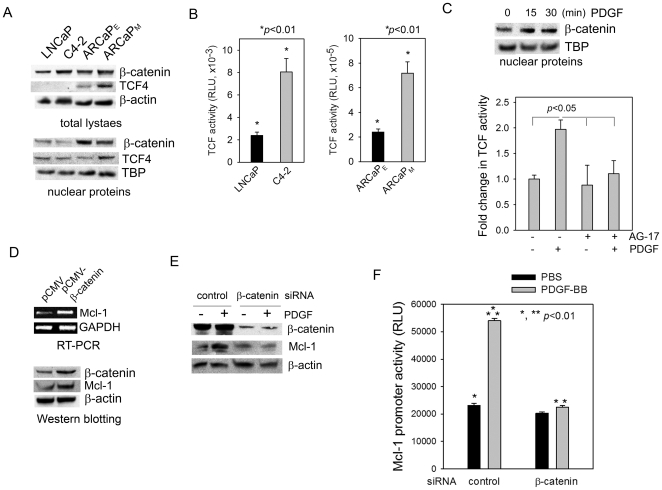
β-catenin mediates PDGF regulation of Mcl-1 expression in PCa cells. (A) Expression profile of β-catenin-TCF signaling components in PCa cells. (B) TCF reporter activity in the LNCaP-C4-2 and ARCaP_E_-ARCaP_M_ cells. (C) Upper panel: The effects of PDGF-BB (20 ng/ml) on the nuclear translocation of β-catenin in ARCaP_M_ cells; Bottom panel: The effects of PDGF-BB (20 ng/ml) on TCF reporter activity in the presence (100 nM) or absence of AG-17. (D) The effects of ectopic expression of β-catenin (72 h) on Mcl-1 expression at both mRNA and protein levels. (E) The effects of β-catenin depletion on PDGF-BB regulation of Mcl-1 expression in ARCaP_M_ cells. The cells were transfected with β-catenin siRNA or control siRNA (30 nM) for 48 h, serum-starved overnight, and incubated in the presence or absence of PDGF-BB (20 ng/ml) for 72 h. (F) The effects of β-catenin depletion on Mcl-1 reporter activity in ARCaP_M_ cells. The cells were transfected with β-catenin or control siRNA (30 nM) for 48 h, and further transfected with a human Mcl-1 reporter for 24 h. Following serum starvation overnight, the cells were incubated in the presence or absence of PDGF-BB (20 ng/ml) for 48 h.

Activation of the β-catenin pathway is a downstream event of PDGF signaling in certain epithelial cancer cells [7], [8], [29]. Western blot analysis found that β-catenin and TCF4, a major β-catenin-interacting transcription factor [30], were differentially expressed in PCa cells (Figure 3A), suggesting a functional β-catenin-TCF4 signaling in these cells. In fact, an artificial TCF promoter was activated in both the LNCaP-C4-2 and ARCaP_E_-ARCaP_M_ cell lineages, and the reporter activities appeared to be associated with increased *in vivo* metastatic potential in C4-2 and ARCaP_M_ cells (Figure 3B). It is worth noting that both β-catenin and TCF4 were substantially presented in the nucleus of ARCaP_E_ and ARCaP_M_ cells (Figure 3A, low panel), which exhibited markedly higher basal TCF activities than either LNCaP or C4-2 cells (by ∼100-fold) (Figure 3B).

Upon PDGF-BB treatment, the nuclear presence of β-catenin was rapidly increased in ARCaP_M_ cells (Figure 3C, upper panel). Consistently, TCF reporter activity was also significantly increased following PDGF-BB stimulation, which was attenuated by the pre-treatment with AG-17 (Figure 3C, bottom panel). These data indicated that PDGF-BB activated β-catenin signaling in a PDGFR-dependent manner.

To investigate the role of β-catenin in the regulation of Mcl-1 expression, ARCaP_M_ cells were transiently transfected with a construct expressing wild-type β-catenin. RT-PCR and Western blot analyses showed that ectopic epression of β-catenin increseased Mcl-1 at both mRNA and protein levels (Figure 3D). In contrary, β-catenin depletion using a siRNA pool efficiently inhibited both the basal expression of Mcl-1 and its induction by PDGF-BB (Figure 3E). Consistently, whereas PDGF-BB significantly induced the luciferase activity of a full-length human Mcl-1 promoter in ARCaP_M_ cells transfected with non-targeting control siRNAs, this effect was abrogated by transient depletion of endogeneous β-catenin (Figure 3F). These results suggested that activation of β-catenin signaling may be sufficient and required for Mcl-1 expression in PCa cells.

### PDGF activates p68-β-catenin signaling in PCa cells

**Figure 4:**
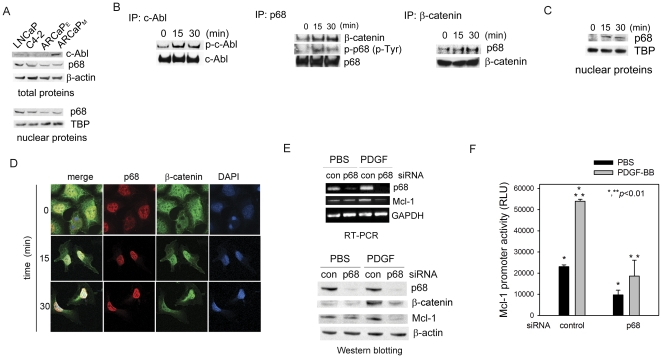
PDGF-BB activates the c-Abl-p68-β-catenin signaling cascade in PCa cells. (A) Expression of c-Abl and p68 in PCa cells. (B) Left panel: The effects of PDGF-BB (20 ng/ml) on the phosphorylation of c-Abl; middle panel: The effects of PDGF-BB (20 ng/ml) on the phosphorylation of p68 and the expression of β-catenin in the p68 immunoprecipitates in ARCaP_M_ cells. Phosphorylation of p68 on the tyrosine sites was detected using a pan-phosphorylated-Tyr antibody; right panel: The effects of PDGF-BB (20 ng/ml) on the expression of p68 in the β-catenin immunoprecipitates in ARCaP_M_ cells. (C) The effects of PDGF-BB (20 ng/ml) on the nuclear translocation of p68 in ARCaP_M_ cells. (D) Confocal microscopy analysis of the effects of PDGF-BB on the co-localization of β-catenin and p68 in the nucleus in a time course experiment in ARCaP_M_ cells. (E) The effects of p68 depletion on PDGF regulation of Mcl-1 in ARCaP_M_ cells. The cells were transfected with p68 or control siRNA (30 nM) for 48 h, serum-starved overnight, and incubated in the presence or absence of PDGF-BB (20 ng/ml) for 24 h (upper panel) or 72 h (bottom panel). Upper panel: RT-PCR analysis of mRNA expression of p68 and Mcl-1; bottom panel: Western blot analysis of protein expression of p68, β-catenin and Mcl-1. (F) The effects of p68 depletion on Mcl-1 reporter activity in ARCaP_M_ cells. The cells were transfected with p68 or control siRNA (30 nM) for 48 h, and further transfected with human Mcl-1 reporter for 24 h. Following serum starvation overnight, the cells were incubated in the presence or absence of PDGF-BB (20 ng/ml) for 48 h.

We investigated whether a c-Abl-p68-dependent pathway is involved in the PDGF activation of β-catenin signaling in PCa cells [7]. Western blot analyses found that c-Abl and p68 were differentially expressed in PCa cells (Figure 4A). Upon PDGF-BB treatment, tyrosine phosphorylation of c-Abl and p68 were rapidly activated, as evidenced by immunoprecipitation-immunoblotting assays (Figure 4B, left and middle panels). Importantly, the presence of β-catenin in p68 immunoprecipitates was also increased in a time-dependent manner, suggesting an enhanced physical association between β-catenin and p68 proteins (Figure 4B, middle panel), which was further confirmed by a reciprocal immunoprecipitation experiment (Figure 4B, right panel). In fact, PDGF-BB induced rapid nuclear translocation of p68 within 30 min (Figure 4C), which was associated with increased co-localization of p68 and β-catenin in the nucleus (Figure 4D). These data indicated that PDGF-BB could activate the c-Abl-p68 cascade and subsequent β-catenin signaling in PCa cells.

To examine whether p68 is required for the regulation of Mcl-1 expression, ARCaP_M_ cells were transfected with p68 siRNA or control siRNA, and analyzed for the expression of Mcl-1 at the mRNA and protein levels. As shown in Figure 4E, depletion of p68 inhibited endogeneous β-catenin and effectively attenuated PDGF-BB induction of Mcl-1 expression. Consistently, Mcl-1 promoter activity was significantly inhibited by the treatment with p68 siRNA in ARCaP_M_ cells, either with or without the presence of PDGF-BB in the cultures (Figure 4F). These data indicated an indispensible function of p68 in the regulation of Mcl-1 in PCa cells.

### PDGF-BB promotes protein interaction between β-catenin and HIF-1α in PCa cells

**Figure 5:**
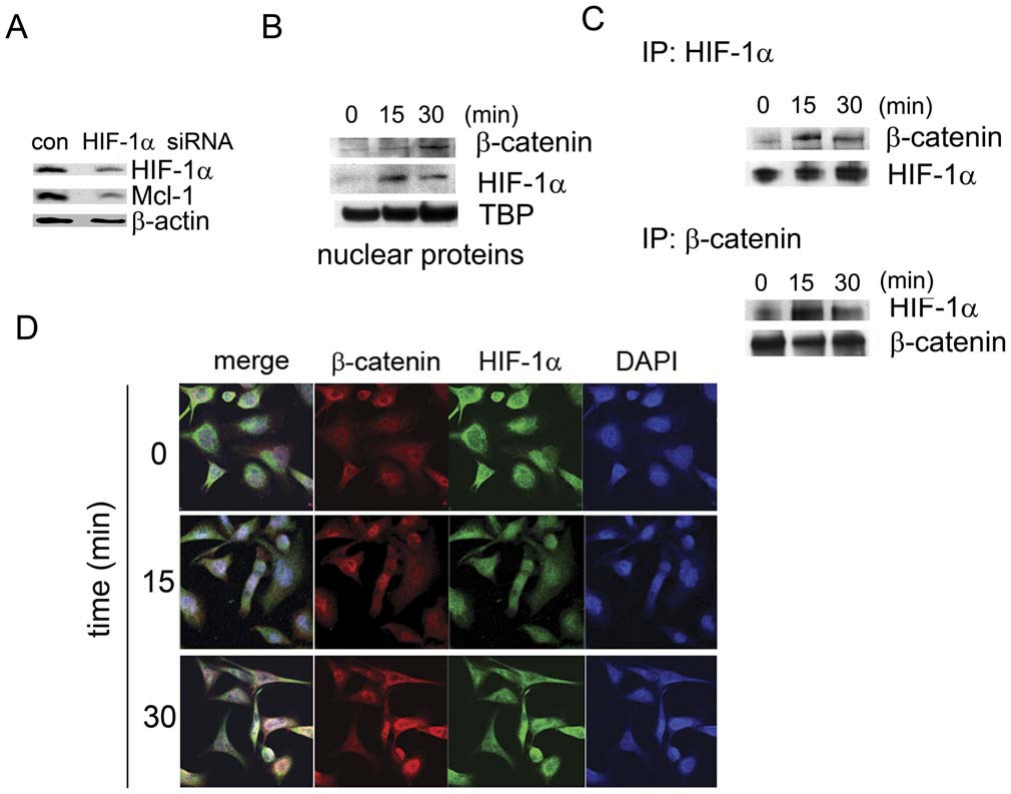
PDGF-BB promotes protein interaction between β-catenin and HIF-1α in PCa cells. (A) The effects of HIF-1α depletion on Mcl-1 expression in ARCaP_M_ cells. The cells were transfected with HIF-1α or control siRNA (30 nM) for 72 h, and analyzed for Mcl-1 expression by immunoblotting. (B) Western blot analysis of the effects of PDGF-BB (20 ng/ml) on the nuclear translocation of β-catenin and HIF-1α in ARCaP_M_ cells. (C) Co-immunoprecipitation assays of the effects of PDGF-BB (20 ng/ml) on the interaction between β-catenin and HIF-1α in the nucleus in ARCaP_M_ cells. (D) Confocal microscopy of the effects of PDGF-BB (20 ng/ml) on the co-localization of β-catenin and HIF-1α in the nucleus in ARCaP_M_ cells.

Our previous studies demonstrated an important role of HIF-1α in bone metastatic PCa cells [25]. Interestingly, transfection of a HIF-1α-specific siRNA significantly reduced Mcl-1 protein expression in ARCaP_M_ cells (Figure 5A), suggesting that HIF-1α may be required for Mcl-1 regulation in PCa cells. To examine whether PDGF-BB could induce physical interaction between HIF-1α and β-catenin, nuclear proteins were prepared from ARCaP_M_ cells treated with PDGF-BB for varying times. Western blot analysis found that both HIF-1α and β-catenin were rapidly increased in the nucleus (Figure 5B). A co-immunoprecipitation assay showed that in response to PDGF-BB stimulation, nuclear presence of β-catenin rapidly increased in the HIF-1α immunoprecipitates (Figure 5C, upper panel). Reciprocal co-immunoprecipitation with an anti-β-catenin antibody confirmed an increased association of nuclear HIF-1α with β-catenin following PDGF-BB treatment (Figure 5C, bottom panel). The enhanced co-localization of β-catenin and HIF-1α proteins was further demonstrated by confocal microscopy, which appeared to acheive the maximum intensity at 30 min upon PDGF-BB stimulation (Figure 5D). These results indicated that in repsonse to PDGF-BB stimulation, β-catenin physically interacts with HIF-1α in the nucleus, which may lead to the activation of Mcl-1 transcription in PCa cells.

### A putative HRE motif is required for PDGF-BB activation of Mcl-1 promoter

**Figure 6:**
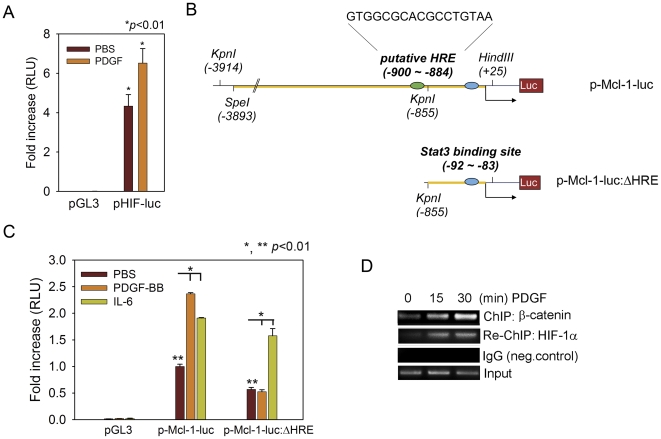
A putative HRE site is required for PDGF-BB activation of Mcl-1 promoter. (A) The effects of PDGF-BB on the HIF-1 reporter activity in ARCaP_M_ cells. The cells were transiently transfected with HIF-1 reporter or pGL3 for 24 h, serum-starved and incubated in the presence or absence of PDGF-BB (20 ng/ml) for 48 h. (B) Schematic diagram of human Mcl-1 promoter and its deletion mutation at the putative HRE site. (C) The effects of deleting the putative HRE site on PDGF regulation of Mcl-1 promoter activity in ARCaP_M_ cells. IL-6 (200 ng/ml) was included as the positive control. (D) ChIP-Re-ChIP assay of the effects of PDGF-BB treatment (20 ng/ml) on the binding of β-catenin and HIF-1α to human Mcl-1 promoter region in ARCaP_M_ cells.

HIF-1α binds to the HRE *cis*-elements within the promoters of hypoxia-responsive genes and regulates their expression [31]. We examined whether PDGF-BB-induced nuclear accumulation of HIF-1α was associated with the activation of HRE-dependent transcription. In ARCaP_M_ cells, PDGF-BB treatment significantly increased luciferase expression driven by an artificial HRE promoter (pHIF-luc) (Figure 6A). Interestingly, a putative HRE motif was identified within human Mcl-1 promoter region, which is located between -900 and -884 nucleotides at the 5′-upstream of transcription start site [24]. To investigate the potential role of this *cis*-element in PDGF regulation of Mcl-1 transcription, we characterized a deletion mutant of the putative HRE motif using human Mcl-1 promoter region as the template (Figure 6B). The resulting reporter construct (p-Mcl-1-Luc: ΔHRE), or the luciferase reporter driven by the full-length Mcl-1 promoter (p-Mcl-1-Luc), was transiently expressed in ARCaP_M_ cells respectively, and treated with PDGF-BB or PBS. IL-6, which has been shown to activate Mcl-1 transcription in PCa and cholangiocarcinoma cells through a signal transducer and activator of transcription 3 (Stat3)-dependent mechanism [32], [33], was included as the positive control. Luciferase activity assay showed that PDGF-BB induced the activation of p-Mcl-1-Luc promoter to a greater degree than IL-6 in ARCaP_M_ cells. Significantly, deletion of the HRE motif not only reduced the basal activity of Mcl-1 promoter, but also abrogated the inductive effects of PDGF-BB on reporter activity. In contrary, p-Mcl-1-Luc: ΔHRE, containing a Stat3-binding sequence at position between −92 and −83 [32], remained activated upon IL-6 treatment (Figure 6C). A similar effect of HRE deletion on the differential response of Mcl-1 promoter to PDGF-BB and IL-6 was also observed in C4-2 cells (Supplemental Figure S2). These data indicated that the putative HRE *cis*-element is required for PDGF-BB activation of Mcl-1 expression in PCa cells.

### PDGF-BB promotes the binding of both β-catenin and HIF-1α to Mcl-1 promoter region

We investigated whether PDGF-BB promoted specific binding of both β-catenin and HIF-1α to Mcl-1 promoter by a sequential ChIP assay. Fractionated chromatin from controls and PDGF-BB-treated ARCaP_M_ cells was firstly immunoprecipitated with a β-catenin antibody, and the precipitates were subjected to a re-ChIP assay with an HIF-1α antibody. From the isolated DNA, a 151-bp fragment containing the HRE region on the Mcl-1 promoter could be amplified from the re-ChIP precipitates. Upon PDGF-BB stimulation, a considerable increase in the binding of both β-catenin and HIF-1α to the HRE region was observed (Figure 6D). These results demonstrated that PDGF-BB could facilitate the association of Mcl-1 promoter with a transcriptional complex consisting of β-catenin and HIF-1α in PCa cells.

## Discussion

**Figure 7:**
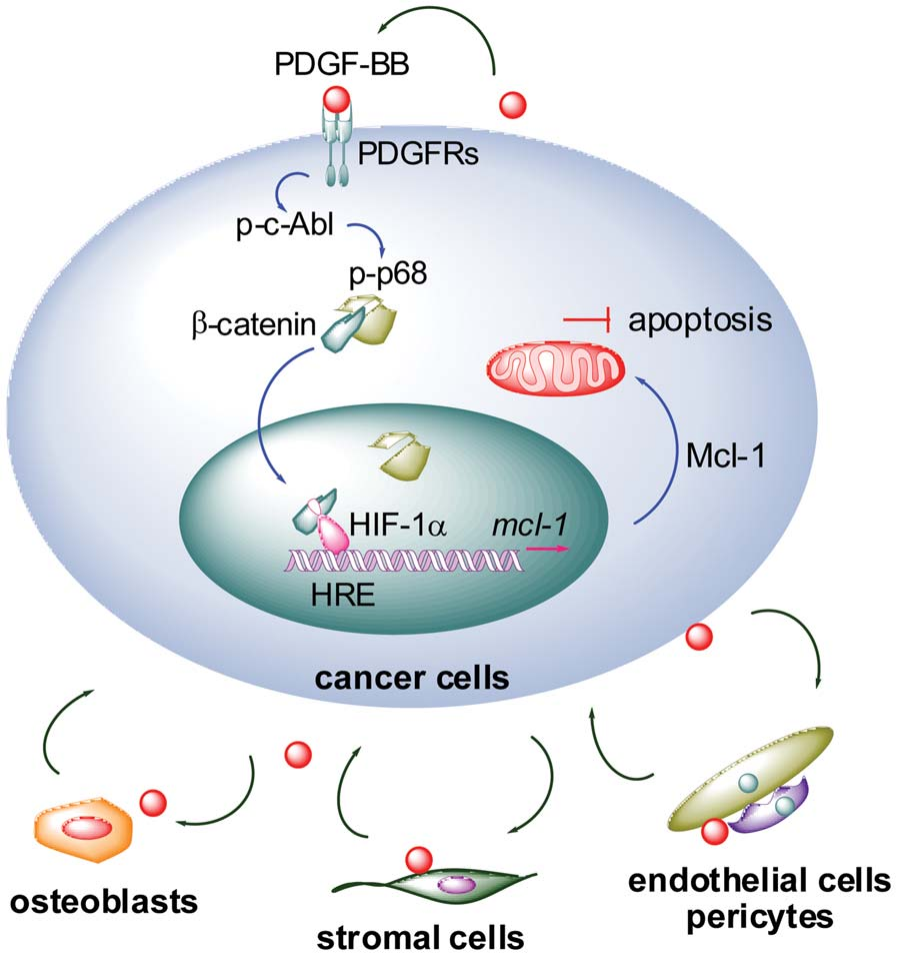
A proposed model for PDGF-BB regulation of Mcl-1 expression in PCa cells. The engagement of PDGF-BB to PDGFR dimers activates the c-Abl-p68 cascade, which subsequently stablizes β-catenin and promotes its nuclear translocation. In the nucleus, interaction between β-catenin and HIF-1α increases the binding of HIF-1α to the HRE site within Mcl-1 promoter, thereby activating the transcription of Mcl-1 gene. Upregulation of Mcl-1 antagonizes apoptotic signals and confers survival advantages to metastatic PCa cells. Furthermore, tumor-derived and locally expressed PDGF may mediate the interactions between PCa and bone microenvironment. Co-targeting the PDGF signaling in PCa cells (autocrine) and microenvironment (paracrine) could provide a new strategy to disrupt the “vicious cycle” and efficaciously treat metastatic PCa.

In this study, we uncovered the PDGF-Mcl-1 signaling as a crucial survival mechanism in PCa cells (Figure 7). For the first time, we demonstrated that: 1) PDGF-BB is a novel regulator of Mcl-1 expression; 2) PDGF-BB activation of autocrine PDGFR signaling promotes the interaction between β-catenin and HIF-1α, presumably through a c-Abl-p68-dependent mechanism; 3) a putative HRE motif is required for the basal expression and PDGF-BB activation of Mcl-1 promoter; and 4) inhibition of the PDGFR-Mcl-1 signaling using a small-molecule inhibitor AG-17 could activate apoptotic response in metastatic PCa cells. These results support that targeting PDGF-Mcl-1 pathway may provide a novel strategy for treating PCa metastasis.

Activation of PDGFR signaling may be coupled with multiple downstream pathways in the regulation of cell growth, proliferation, migration and survival [3]. In tumor-associated endothelial and fibroblast stromal cells, PDGF has been shown to activate Akt- and MAPK-dependent survival mechanisms [34], [35], [36], [37]. Yet, it remains elusive on the molecular mechanism by which PDGF exerts its functions in epithelial cancer cells. Recent data have linked autocrine PDGF signaling to the activation of β-catenin pathway. For instance, PDGF-AB was found to induce nuclear β-catenin accumulation via a PI3K-dependent mechanism, thereby protecting hepatocellular carcinoma cells from anoikis during metastatic dissemination [8]. In human colon cancer cells, PDGF-BB induces EMT [7] and upregulates cyclin D1 and c-Myc [38] by activating β-catenin-dependent gene expression. In both cases, PDGF-BB induces the phosphorylation of c-Abl kinase, which subsequently recruits p68, an RNA helicase with ATPase activity, and activates its phosphorylation. Phosphorylated p68 binds β-catenin and promotes its nuclear translocation by displacing Axin from β-catenin and blocking β-catenin degradation, eventually promoting the interaction of β-catenin with TCF/LEF and the assembly of transcription complexes [7]. In this study, we provided molecular evidence demonstrating that in PCa cells that express high basal levels of p68 and β-catenin, PDGF could significantly promote physical interaction and rapid nuclear translocation of p68 and β-catenin. Importantly, p68 depletion in PCa cells led to the inhibition of Mcl-1 expression and induction of apoptosis, as evidenced by the appearance of cleaved PARP (Supplemental Figure S3). These results, for the first time, underscored a critical role of p68 in the regulation of PCa cell survival. Interestingly, a recent study demonstrated that p68 is actually a novel coactivator of androgen receptor (AR) [39], another transcription factor interacting with β-catenin in certain PCa cells (such as LNCaP and C4-2) [40]. It would be intriguing to further investigate the dynamic interaction between p68, β-catenin and AR, and its biological consequences in these cells. In addition, other pathways may be involved in the PDGF activation of β-catenin signaling. For example, PDGF-BB treatment was found to induce rapid phosphorylation of both Akt and glycogen synthase kinase 3-β (GSK-3β) (Supplemental Figure S4), which may also contribute to the elevated intracellular levels and nuclear accumulation of β-catenin [41].

Our data confirmed a highly active β-catenin/TCF signaling in ARCaP cells and correlated the TCF reporter activity with the *in vivo* metastatic potential (Figure 3A, 3B), indicating these cells could be used as an excellent model system for investigating β-catenin signaling in PCa progression [42]. Though PDGF-BB activated the full-length human Mcl-1 promoter (Figure 3F) in a similar manner to its effect on the luciferase expression driven by an artificial TCF-binding motif (pTOPFlash), it appeared that human Mcl-1 promoter does not contain any consensus sequences of TCF/lymphoid enhancer-binding factor (LEF). These results suggested that certain transcription factor(s), other than TCF, could be responsible for β-catenin activation of Mcl-1 transcription. One of such candidates was cAMP-response element-binding protein (CREB), which has been implicated in the regulation of Mcl-1 expression through the PI-3K/Akt signaling pathway [43] and highly expressed in ARCaP cell lineage [25]. Western blotting analyses, however, could not detect a significant increase in nuclear CREB expression upon PDGF treatment (data not shown), suggesting that CREB may not be involved in the β-catenin-dependent activation of Mcl-1 transcription. Intriguingly, the transcription factor HIF-1α was found to be rapidly increased in the nucleus and physically interact with β-catenin following PDGF-BB stimulation, which may mediate Mcl-1 transcription by binding to HRE site(s) within the promoter. These data are consistent with a previous study showing that β-catenin can switch its binding partner from TCF4 to HIF-1α and enhance HIF-1α-mediated transcription, and this dynamic reassembly of β-catenin with HIF-1α may allow colorectal cancer cells to rapidly adapt to hypoxic stress and survive [44]. It is important to note that unlike the cited work, our studies were performed in normoxic PCa cell cultures. Since ARCaP cells substantially express HIF-1α even under normoxia [25], PDGF may significantly affect the expression of hypoxia-responsive or HRE-containing genes by promoting the interaction between β-catenin and HIF-1α in a Wnt-independent mechanism. Upregulation of Mcl-1, as a consequence, could provide pivotal protection against apoptotic signals during dissemination and colonization when the majority of cancer cells remain under normoxia.

Earlier studies reported high expression of PDGFRs in both localized and metastatic PCa, which could be detected in 88% of primary tumors and 80% of the metastases [10], [45]. However, it remains controversial as to which PDGFR isoforms are expressed in PCa cells and primarily responsible for autocrine PDGF signaling [12], [46], [47]. These conflicting results may partially arise from the potential non-specificity of antibodies used in the cited studies, but more importantly, may reflect the intrinsic heterogeneity of human cancers, especially when at their late-stages. In this study, we were able to detect the expression of both PDGFR isoforms in several established PCa cell lines by RT-PCR and Western blot analyses. Given the fact that both PDGFR-α and -β have been implicated in the progression of bone metastatic PCa [10], [12], [13], [45], [48], our study focused on the function of PDGF-BB since it is the only PDGF isoform that binds all the three receptor dimeric combinations (PDGFR-αα, -ββ and -αβ) with high affinity [2], [49]. To determine which PDGFR isoform is required for PDGF regulation of Mcl-1, we transfected PCa cells with specific siRNAs against PDGFR-α or -β. Interestingly, the single depletion of neither PDGFR-α nor PDGFR-β inhibited Mcl-1 expression in ARCaP_M_ cells, suggesting that the PDGF-BB signal could be transduced via the two independent but complementary receptors to activate Mcl-1 expression in PCa cells expressing both isoforms. Supporting this notion, dual depletion of both receptors simultaneously using a mixture of siRNAs against PDGFR-α and -β effectively inhibited Mcl-1 expression. Alternatively, treatment with AG-17 or imatinib, two pan-PDGFR inhibitors that could inhibit the tyrosine kinase activity of both PDGFR-α and -β, also reduced Mcl-1 levels in ARCaP_M_ cells (Supplemental Figure S5). Furthermore, in PCa cells that predominantly express one PDGFR isoform (for example, PDGFR-α is the major isoform in C4-2 cells; Figure 2A, right panel), it is plausible to expect that inhibition of the isoform alone could affect Mcl-1 expression. Indeed, transfection of PDGFR-α siRNA in C4-2 cells significantly inhibited Mcl-1 (Supplemental Figure S6). These findings support a model that PDGF-BB could activate both PDGFR isoforms in the regulation of Mcl-1 in PCa cells in a context-dependent manner, which may have important implication in the evaluation of PDGFR expression at tissue levels in clinical PCa specimens.

Interaction between PCa and bone microenvironment is crucial to the bone tropism of PCa metastasis, which is identified at autopsy in up to 90% of patients dying from the disease [50]. Tumor-initiated bone resorption promotes the release and activation of multiple growth factors immobilized in bone matrix, including PDGF. These locally expressed and tumor-derived PDGF could activate PDGFR signaling in surrounding stroma (including stromal cells, endothelial cells and pericytes) and promote angiogenesis. As a potent mitogen for osteoblasts, PDGF also significantly contribute to the osteoblastic phenotype of PCa bone metastasis [51]. These effects, taken together, may provide a favorable microenvironment for the survival and outgrowth of bone metastatic PCa. These facts provided rationale for evaluating the potential of treating PCa bone metastasis with small-molecule PDGFR inhibitors. In earlier studies, imatinib sensitized bone marrow stromal and endothelial cells to paclitaxel treatment and significantly suppressed PCa bone metastasis in experimental models [52], [53]. Disappointingly, however, recent clinical trials with imatinib only achieved limited success due to unexpected severe side effects in patients [48]. These observations highlighted the importance of a better understanding of PDGF signaling in bone metastasis PCa. Our study delineated a novel signaling axis that may allow PCa cells to escape apoptosis during dissemination and colonization by activating PDGF-Mcl-1 pathway in metastatic cancer cells. It is plausible to hypothesize that PDGF-BB may be crucial in mediating the “vicious cycle” between tumor and bone microenvironment, not only promoting angiogenesis in surrounding stroma but also sustaining survival in PCa cells (Figure 7). Supporting this model, PDGF-BB was found to be elevated in PC3-MM2 cells implanted in the mouse bone cortex, and interestingly, activated PDGFR-β was only detected in tumor lesions growing adjacent to bone and the tumor-associated endothelium [53], [54]. Given the clinical significance of both PDGF and Mcl-1 in PCa bone metastasis [21], [51], specific targeting of PDGF-Mcl-1 survival pathway in PCa cells (autocrine signaling) and co-targeting of microenvironment (paracrine signaling) could provide a new strategy to disrupt the vicious cycle and efficaciously treat metastatic PCa.

## Supporting Information

Figure S1 **The effects of PDGF-BB on the expression of Mcl-1 at mRNA and protein levels in PCa cells.** (A–C) RT-PCR and qRT-PCR analyses of the time- and dose-dependent effects of PDGF-BB on Mcl-1 mRNA expression in ARCaP_E_ (A), LNCaP (B) and C4-2 (C) cells. The dose was 20 ng/ml in the time course experiments. (D) Western blot analysis of the effects of PDGF isoforms on the expression of Mcl-1 in several PCa cell lines. Treatment: 20 ng/ml, 72 h.
(TIF)

Figure S2 **The effects of deleting the putative HRE site on PDGF regulation of Mcl-1 promoter activity in C4-2 cells.** IL-6 (200 ng/ml) was included as the positive control.
(TIF)

Figure S3 **The effects of p68 siRNA on the expression of cleaved PARP, an indicator of apoptosis in PCa cells.** ARCaP_M_ cells were transfected with p68 or control siRNA (30 nM) for 48 h, serum-starved overnight, and incubated in the presence or absence of PDGF-BB (20 ng/ml) for 72 h.
(TIF)

Figure S4 **The effect of PDGF-BB on the Akt-GSK-3β cascade in PCa cells.** PDGF-BB treatment (20 ng/ml) in ARCaP_M_ cells increased the phosphorylation of Akt and GSK-β at serine residues.
(TIF)

Figure S5 **Imatinib, a small-molecule inhibitor of PDGFR signaling, inhibits Mcl-1 protein expression in PCa cells.** ARCaP_M_ cells were treated with 10 µM imatinib for varying times, western blotting was then performed.
(TIF)

Figure S6 **Depletion of PDGFR-α abrogates PDGF-BB induction of Mcl-1 in C4-2 cells.** The cells were transfected with PDGFR-α or control siRNA (30 nM) for 48 h, serum-starved overnight, and incubated in the presence or absence of PDGF-BB (20 ng/ml) for 72 h.
(TIF)

Table S1 **Antibodies used in this study.**
(PDF)

Table S2 **Primers for PCR and RT-PCR.**
(PDF)
