# Supplementary figures and images for: PDGF Upregulates Mcl-1 Through Activation of β-Catenin and HIF-1α-Dependent Signaling in Human Prostate Cancer Cells
Source: PLoS One. 2012 Jan 20;7(1):e30764. doi: 10.1371/journal.pone.0030764 (PMC3262835; doi:10.1371/journal.pone.0030764)

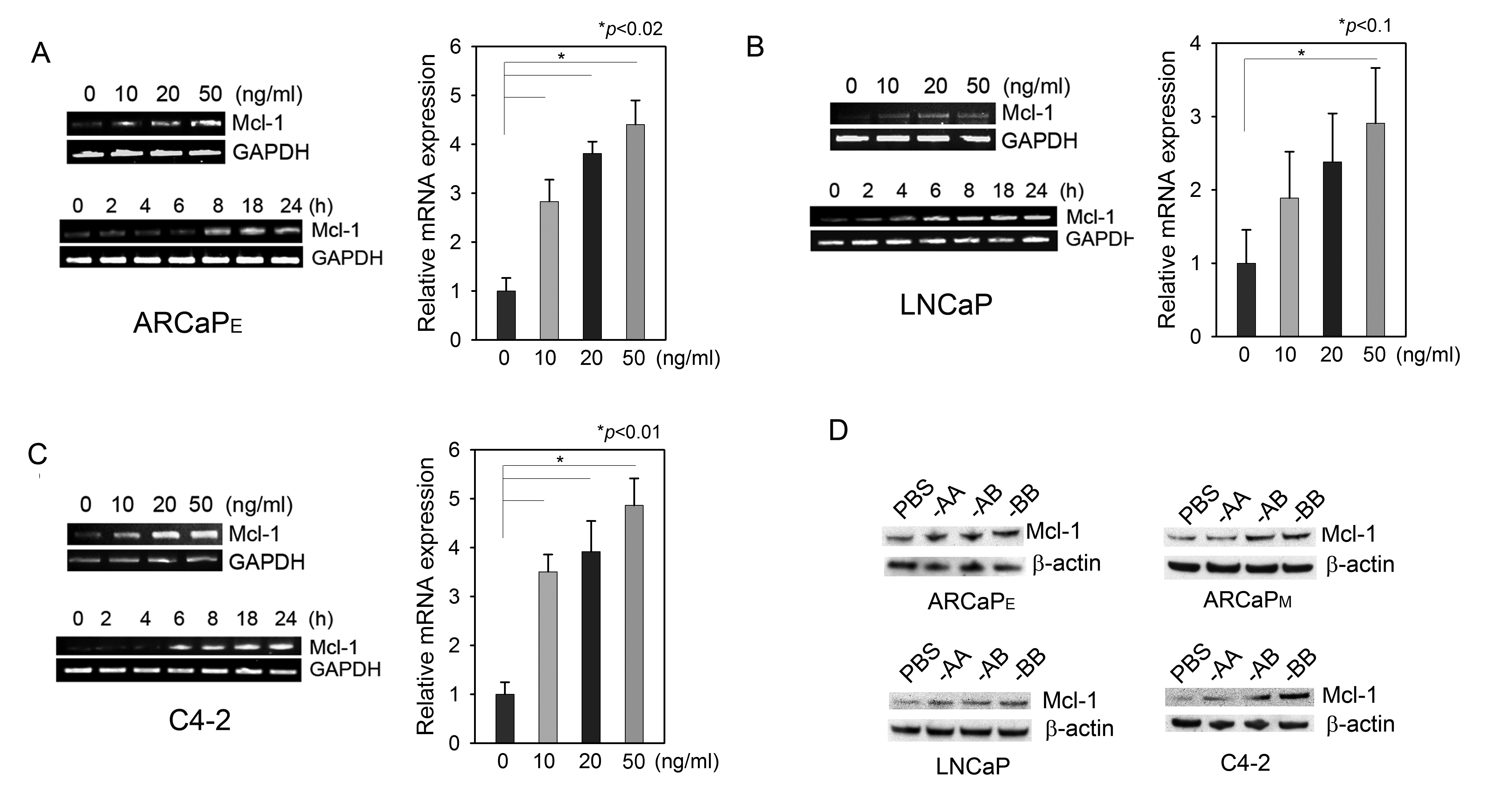

Supplement: Figure S1 — The effects of PDGF-BB on the expression of Mcl-1 at mRNA and protein levels in PCa cells. (A–C) RT-PCR and qRT-PCR analyses of the time- and dose-dependent effects of PDGF-BB on Mcl-1 mRNA expression in ARCaPE (A), LNCaP (B) and C4-2 (C) cells. The dose was 20 ng/ml in the time course experiments. (D) Western blot analysis of the effects of PDGF isoforms on the expression of Mcl-1 in several PCa cell lines. Treatment: 20 ng/ml, 72 h. (TIF) [file pone.0030764.s001.tif]

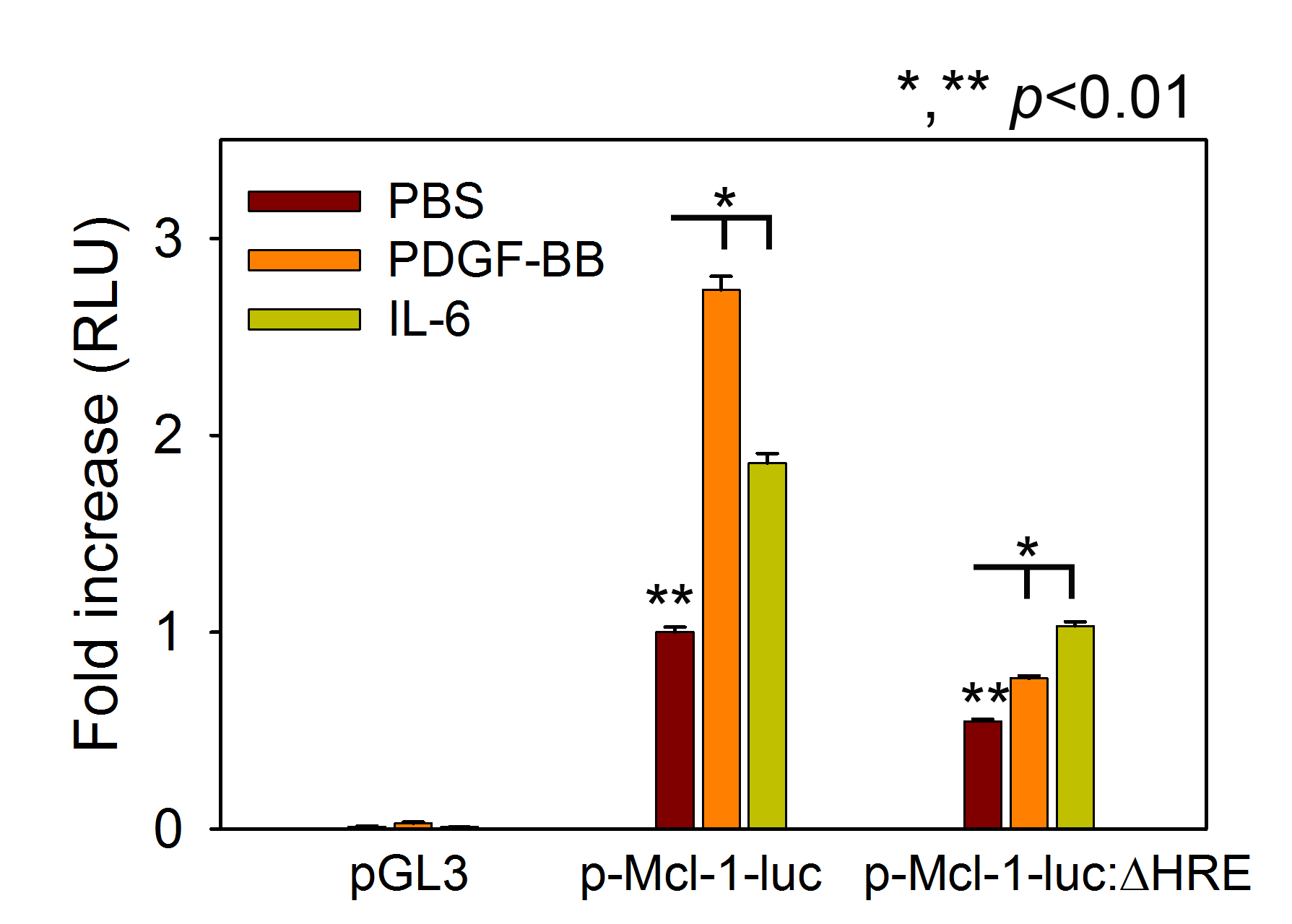

Supplement: Figure S2 — The effects of deleting the putative HRE site on PDGF regulation of Mcl-1 promoter activity in C4-2 cells. IL-6 (200 ng/ml) was included as the positive control. (TIF) [file pone.0030764.s002.tif]

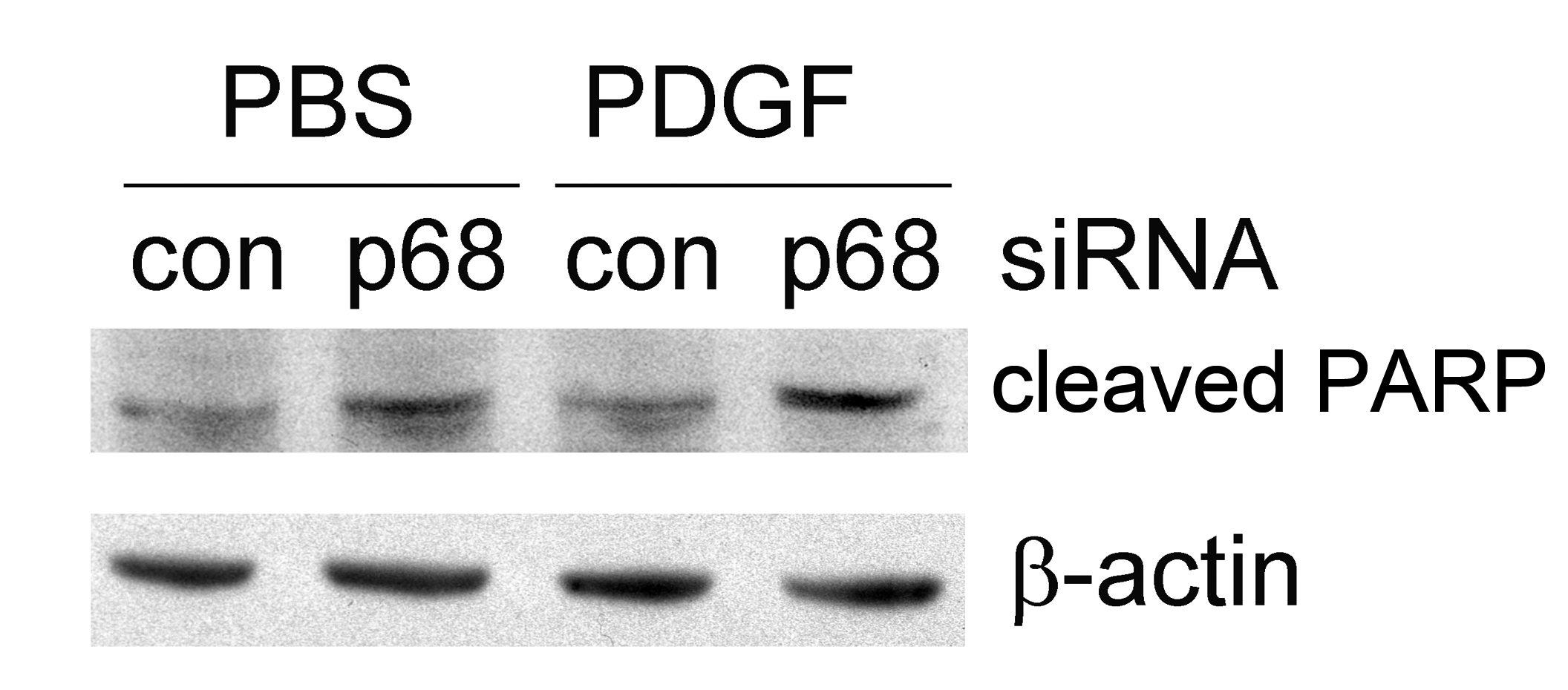

Supplement: Figure S3 — The effects of p68 siRNA on the expression of cleaved PARP, an indicator of apoptosis in PCa cells. ARCaPM cells were transfected with p68 or control siRNA (30 nM) for 48 h, serum-starved overnight, and incubated in the presence or absence of PDGF-BB (20 ng/ml) for 72 h. (TIF) [file pone.0030764.s003.tif]

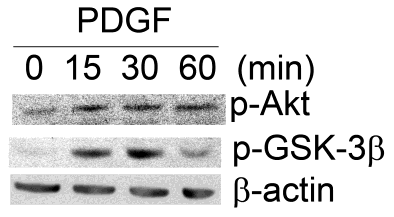

Supplement: Figure S4 — The effect of PDGF-BB on the Akt-GSK-3β cascade in PCa cells. PDGF-BB treatment (20 ng/ml) in ARCaPM cells increased the phosphorylation of Akt and GSK-β at serine residues. (TIF) [file pone.0030764.s004.tif]

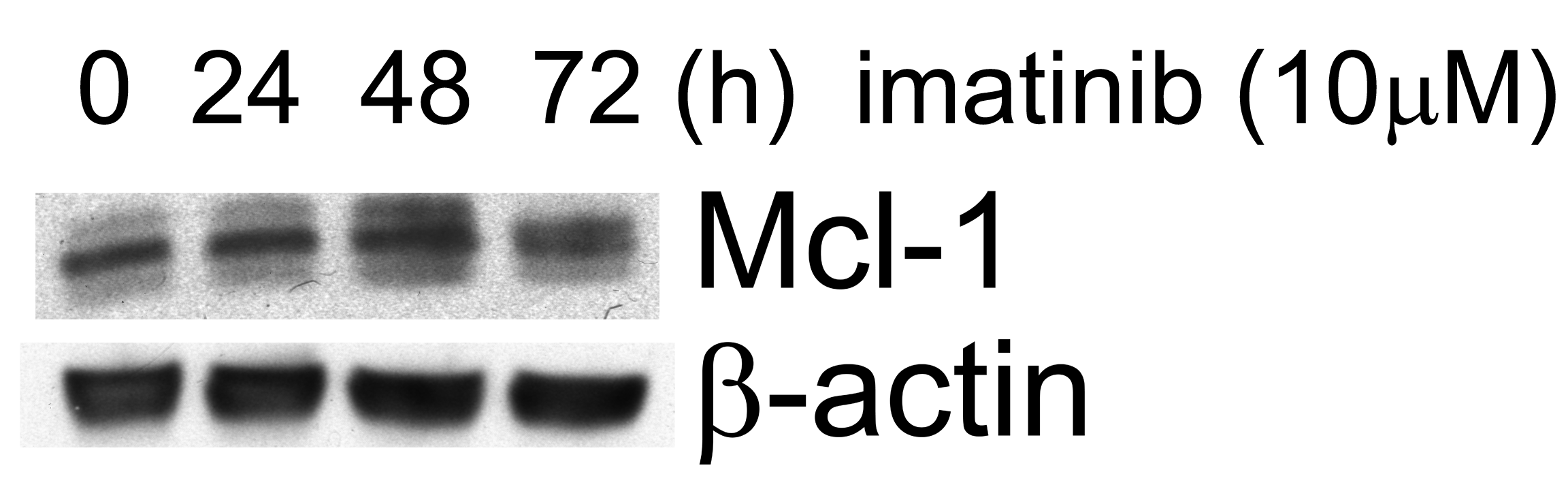

Supplement: Figure S5 — Imatinib, a small-molecule inhibitor of PDGFR signaling, inhibits Mcl-1 protein expression in PCa cells. ARCaPM cells were treated with 10 µM imatinib for varying times, western blotting was then performed. (TIF) [file pone.0030764.s005.tif]

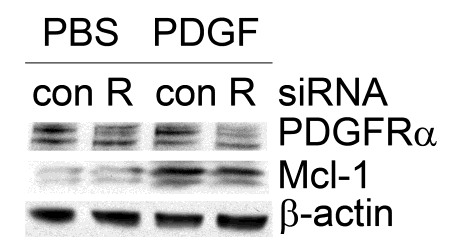

Supplement: Figure S6 — Depletion of PDGFR-α abrogates PDGF-BB induction of Mcl-1 in C4-2 cells. The cells were transfected with PDGFR-α or control siRNA (30 nM) for 48 h, serum-starved overnight, and incubated in the presence or absence of PDGF-BB (20 ng/ml) for 72 h. (TIF) [file pone.0030764.s006.tif]
