# Supplementary material for: PDGF Upregulates Mcl-1 Through Activation of β-Catenin and HIF-1α-Dependent Signaling in Human Prostate Cancer Cells
Source: PLoS One. 2012 Jan 20;7(1):e30764. doi: 10.1371/journal.pone.0030764 (PMC3262835; doi:10.1371/journal.pone.0030764)
Supplement: Table S1 — Antibodies used in this study. (PDF) [file pone.0030764.s007.pdf]

**Antibody**	**Host Animal**	**Company**	**Cat. No.**	**Dilution**
β-actin	Mouse	Sigma	A 5441	1-1000
β -catenin	Mouse	BD Transduction	610154	1-500
β-catenin (H-102)	Rabbit	Santa Cruz	sc-7199	1-500
Bcl-2 (N-19)	Rabbit	Santa Cruz	*sc-492*	*1-100*
c-Abl(24-11)	Mouse	Santa Cruz	sc-23	1-200
E-cadherin (H-108)	Rabbit	Santa Cruz	sc-7870	1-500
HIF-1α	Rabbit	Millipore	07-628	1-200
HIF-1α	Mouse	BD Transduction	610958	1-200
Mcl-1	Rabbit	Santa Cruz	sc-819	1-500
p68 RNA Helicase (H-144)	Rabbit	Santa Cruz	sc-32858	1-200
PARP	Rabbit	Cell Signaling	9542	1-500
PDGFR-α (951)	Rabbit	Santa Cruz	sc-431	1-100
PDGFR-β	Rabbit	Santa Cruz	sc-339	1-100
TCF-4 (N-20)	Goat	Santa Cruz	sc-8631	1-100
TFIID (TBP) (N-12)	Rabbit	Santa Cruz	sc-204	1-200
Phospho-c-Abl (Tyr 245)	Rabbit	Cell Signaling	2861	1-500
Phospho-PDGFR-α (Tyr754)	Rabbit	Cell Signaling	2992	1-1000
Phospho-PDGFR-β (Tyr751)	Mouse	Cell Signaling	3166	1-1000
Phospho-Tyrosine (P-Tyr-100)	Mouse	Cell Signaling	9411	1-1000
Normal IgG	Rabbit	Cell Signaling	2729	
Normal IgG	Mouse	Millipore	12-371	
