# Supplementary material for: PDGF Upregulates Mcl-1 Through Activation of β-Catenin and HIF-1α-Dependent Signaling in Human Prostate Cancer Cells
Source: PLoS One. 2012 Jan 20;7(1):e30764. doi: 10.1371/journal.pone.0030764 (PMC3262835; doi:10.1371/journal.pone.0030764)
Supplement: Table S2 — Primers for PCR and RT-PCR. (PDF) [file pone.0030764.s008.pdf]

Gene	Sequence (5'-3')	Annealing Temp	Cycles
PDGFR-α (forward)	AAATGGGTGCTAAATTGATTGG		
PDGFR-α (reverse)	GCACATCTTTAGCAGGAGCC	65	35
PDGFR-β (forward)	GTGCTCACCATCATCTCCCT		
PDGFR-β (reverse)	ACTCAATCACCTTCCATCGG	55	40
PDGF-A (forward)	ACACGAGCAGTGTCAAGTGC		
PDGF-A (reverse)	CCTGCAGTATTCCACCTTGG	65	35
PDGF-B (forward)	AGATCGAGATTGTGCGGAAG		
PDGF-B (reverse)	CAGCTGCCACTGTCTCACAC	55	40
PDGF-C (forward)	GCCAGGTTGTCTCCTGGTTA		
PDGF-C (reverse)	TGCTTGGGACACATTGACAT	65	35
PDGF-D (forward)	CCCAGGAATTACTCGGTCAA		
PDGF-D (reverse)	ACAGCCACAATTTCCTCCAC	65	35
HRE (forward)	AGGTCACTTGAGGCCATGAG		
HRE (reverse)	CACGTTCAGACGATTCGGTA	59	40
GAPDH (forward)	GTCAGTGGTGGACCTGACCT		
GAPDH (reverse)	AGGGTCTACATGGCAACTG	65	23
Mcl-1 (forward)	GAGGAGGAGGAGGACGAGTT		
Mcl-1 (reverse)	GTCCCGTTTTGTCCTTACGA	65	23
